# Assessing the burden of pregnancy-associated malaria under changing transmission settings

**DOI:** 10.1186/1475-2875-8-245

**Published:** 2009-10-28

**Authors:** Mario Recker, Menno J Bouma, Paul Bamford, Sunetra Gupta, Andy P Dobson

**Affiliations:** 1 Department of Zoology, University of Oxford, Oxford OX1 3PS, UK; 2 Department of Infectious and Tropical Diseases, London School of Hygiene and Tropical Medicine, University of London, London WC1E 7HT, UK; 3 Department of Ecology and Evolutionary Biology, Princeton University, Princeton, NJ 08544, USA

## Abstract

**Background:**

The clinical presentation of pregnancy-associated malaria, or PAM, depends crucially on the particular epidemiological settings. This can potentially lead to an underestimation of its overall burden on the female population, especially in regions prone to epidemic outbreaks and where malaria transmission is generally low.

**Methods:**

Here, by re-examining historical data, it is demonstrated how excess female mortality can be used to evaluate the burden of PAM. A simple mathematical model is then developed to highlight the contrasting signatures of PAM within the endemicity spectrum and to show how PAM is influenced by the intensity and stability of transmission.

**Results:**

Both the data and the model show that maternal malaria has a huge impact on the female population. This is particularly pronounced in low-transmission settings during epidemic outbreaks where excess female mortality/morbidity can by far exceed that of a similar endemic setting.

**Conclusion:**

The results presented here call for active intervention measures not only in highly endemic regions but also, or in particular, in areas where malaria transmission is low and seasonal.

## Background

Malaria during pregnancy poses a significant threat to both the mother and unborn child. For the mother, it increases the risk of illness, severe anaemia and death; for the unborn child it increases the risk of intra-uterine growth retardation and low birth weight, spontaneous abortion and stillbirth (reviewed in [1]). Numerous epidemiological studies have highlighted the various aspects of malaria during pregnancy both in highly endemic regions (mostly from sub-Saharan Africa) and in regions where malaria transmission is low and sporadic (mainly from Asia) (see e.g. [1-4]), and it has become clear that the pathology of malaria during pregnancy and its evaluation are highly dependent on the particular epidemiological setting, due to differences in acquired immunity in women reaching child-bearing age.

Acquired immunity to *Plasmodium falciparum*, the most virulent agent of human malaria, is a gradual process by which individuals build up a repertoire of protective immune responses over years of repeated exposure. Although sterilizing immunity might never be attained, people living in malaria endemic areas seem to acquire protection against clinical malaria after a certain period of exposure [5,6] and to severe disease after only a few infections [7]. In regions of sustained transmission, most women of child-bearing age are, therefore, clinically immune against the disease. Malarial infections during pregnancy in these regions are usually associated with placental sequestration of parasites adhering to glycosaminoglycan chondroitin sulphate A (CSA) through the expression of *var2csa *[8-11], a member of the *var *gene family which encodes the immunodominant surface antigens and virulence factors PfEMP1 (*P.falciparum *membrane protein 1); this phenomenon is also known as pregnancy-associated malaria or PAM.

Despite the vast overall diversity of *var *genes, serum samples from women of different geographical backgrounds have shown that the antigens expressed in infected placentas are highly conserved [12,13]. As a consequence, immunity against PAM is rapid: usually after one or two episodes, women will be protected during subsequent pregnancies [14]. This is also supported by field studies from highly endemic regions showing that PAM predominantly affects primigravidae, i.e. women in their first pregnancy, rather than multigravidae. This epidemiological picture does not, however, extend to low transmission areas where primi- and multigravid women can be equally affected by PAM. Also, pregnant women from regions with low and unstable transmission invariably become symptomatic upon infection, and as a consequence it is harder to differentiate between PAM and normal, i.e. non-PAM, malaria unless placental samples are examined and reported; this, however, is rarely the case in the published literature.

The assessment of the extra burden caused by PAM, therefore, has to take into account the particular transmission setting and is further complicated by at least two important factors. Firstly, women from highly endemic regions with placental malaria rarely show symptoms of disease yet can develop life-threatening anaemia; this can potentially lead to substantial underreporting of maternal and foetal mortality due to placental malaria. Secondly, the rates of mortality which can be attributed to PAM in low transmission settings are hard to assess not only because of the additional threat of clinical disease due to inadequate levels of protection but also because studies rarely report the regions' equivalent (age- and exposure adjusted) mortality rates for men or non-pregnant women. Crucially also, there is still a great need to evaluate the magnitude of the extra burden of PAM, and malaria during pregnancy in general, during epidemic outbreaks.

In this paper, historical data from the Punjab region in former British India is re-examined and used to demonstrate how to qualitatively assess the magnitude of PAM in an epidemic transmission setting by means of excess female mortality. The influence of the mode and intensity of transmission is then investigated by means of a simple mathematical model. Both the data and the model clearly demonstrate that epidemic malaria, especially when intermitted by periods of very low transmission, can have devastating effects on the female population. This calls for active intervention measures not only in highly endemic but also, or even in particular, in regions of unstable malaria transmission and those prone to epidemic outbreaks.

## Methods and Results

### Historical data

**Figure 1:**
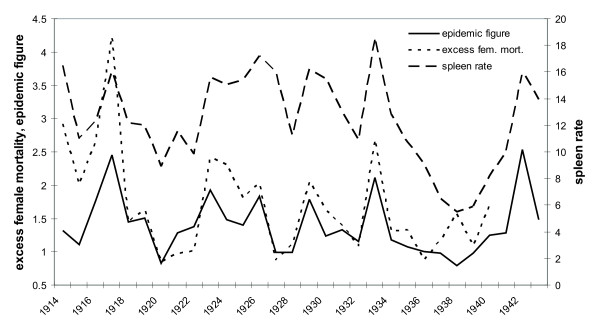
**Spleen rate, Epidemic Figure (EF), and relative female mortality of all age groups between 1914 and 1943 for all districts in the Punjab**. Relative female mortality (as % × 10, dotted line), defined as the ratio of female to male mortalities, correlates well with both malaria indices spleen rate (dashed line) and Epidemic Figure (solid line). The values of the EF and relative female mortality for 1918, during the influenza epidemic, were estimated, based on the linear regression results with the spleen rate (r = 0.76 and r = 0.64 respectively).

Malaria in the semi-arid Punjab province of former British India, currently the Punjab province of Pakistan and the Punjab and Haryana States of India, showed severe epidemics associated with excess rainfall (Annual reports of the Director of Public Health, Punjab 1902-1940, [15,16]). Before the discovery of the malaria parasite and when microscopically confirmed diagnosis was not readily available, fever deaths during the malaria season (October to December) have been used to estimate malaria mortality. The "Epidemic Figure", a measure of the annual (seasonal) intensity of transmission was introduced to assist time series analysis. This ratio of fever deaths during the malaria season (Oct-Dec) and deaths during non-malariaous months was devised to minimize the effects of administrative changes and qualities of the health system over time. Spleen rates surveys, the percentage of school children under the age of 10 with a palpable spleen, were introduced in the Punjab in 1914 to help together with rainfall monitoring identification of areas at risk of epidemics [17]. Figure 1 presents the spleen rate, the Epidemic Figure, and relative female mortality (of all age classes) for all districts in the Punjab for a 30-year period between 1914 and 1943. The graphs clearly indicate a good qualitative agreement between excess female mortality and epidemic figure, as previously observed [18], and the spleen rates conventionally used to assess malaria prevalence. These parameters identify the highly variable rate of malaria (transmission) over the years in this region, including a particularly large epidemic outbreak in 1917; this supports the use of excess female mortality rates as a proxy for qualitatively estimating the additional burden of pregnancy-associated malaria upon the female population.

**Figure 2:**
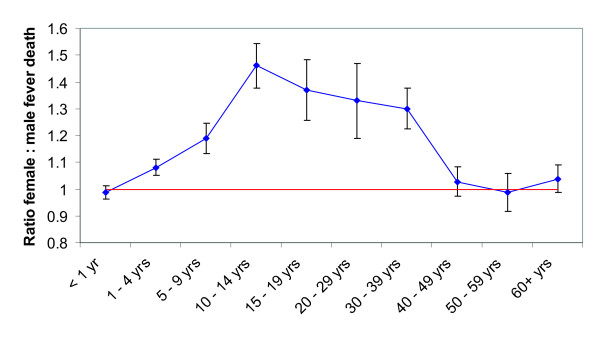
**Relative female to male fever mortality by age group for all Punjab districts (1908 to 1917)**. Excess female fever mortality increase in the 5-9 age group, and remains high during the female reproductive age, only returning to levels comparable to males in the age groups over 40 years. Standard deviations are highest for the 20-29 year age group. Apart from malaria, excess female fever mortality will be associated with perinatal bacterial infections. The slope in the decline of excess mortality between the 10 to 14 and the 30 to 39 year age group could signify a specific pregnancy-associated immunity.

Next, the female population is sub-divided to assess the contributions of different age classes to the level of excess mortality. Figure 2 shows the relative female to male fever mortality by age group for all Punjab districts from 1908 to 1917. This figure clearly shows that that excess female fever mortality peaks in the 10-14 years age group (coinciding with the start of the female reproductive age in historical India) and remains high over the female reproductive years, only returning to levels comparable to males in the age groups over 40 years. This is a clear indication of how malaria affects the group of reproductively active females and primigravidae, i.e. those in the 10-14 years age class, in particular. Furthermore, the slope in the decline of excess mortality between the 10-15 and the 30-39 years age groups could signify the specific pregnancy malaria associated immunity.

**Figure 3:**
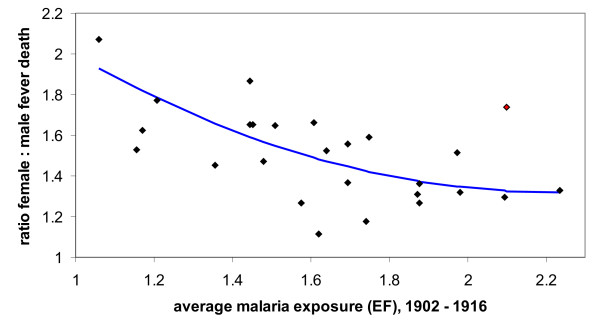
**Relative female fever mortality in all Punjab's 26 districts for the epidemic year 1917 showing the effect of prior exposure on PAM induced morbidity during epidemic outbreaks**. For malaria exposure of the districts prior to 1917, the average annual Epidemic Figures (EF) between 1902 and 1916 for each district were used. One outlier (red diamond) was excluded from the polynomial regression (blue line). Spearman rank coefficient *r *= -0.645, *R*^2 ^= 0.4595, *P *< 0.0005 (with outlier: *r *= -0.519, *R*^2 ^= 0.3725, *P *< 0.007).

To investigate how previous exposure might influence immunity to pregnancy-associated malaria within this region of low and relatively unstable transmission one can plot the female to male fever mortality data from the 1917 epidemic against the epidemic figure as a proxy of the average malaria exposure prior to 1917 (in this case the period 1902 to 1916). The scatter plot in Figure 3 shows how excess female mortality decreases with previous exposure which could be due to an increased level of clinical immunity to malaria that protects women in child-bearing age from severe and symptomatic illness but is also indicative of acquired immunity specifically to placental malaria in that primi- and secundigarvidae are more affected than multigravid women.

Taken together, the data is indicative how malaria and specifically pregnancy-associated malaria affects women in their reproductive age. Although primigravidae are the ones mostly burdened by the disease, multigravid women still contribute significantly to the excess in female mortality, especially during a large epidemic outbreak that is preceded by a long period of low transmission. Next, a simple mathematical framework is developed to qualitatively investigate how the stability and intensity of transmission affects the signature of PAM in terms of excess female mortality (or morbidity in general).

### Mathematical model

**Figure 4:**
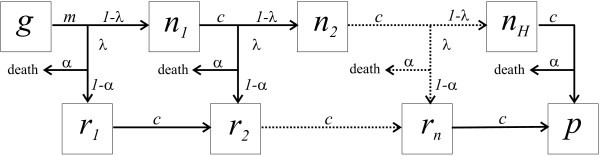
**Flow diagram showing the movements between different compartments within the model**. It is assumed that girls become reproductively active at an average age of 1/*m*. Depending on transmission intensity they contract malaria with probability *λ*, with a proportion a dying as a direct result of the disease. Women who do not contract PAM at their first or subsequent pregnancies remain susceptible and move between different classes of nursing at a rate *c*, where 1/*c *is the average length of time between succeeding pregnancies, until they cease to be reproductively active.

A schematic representation of the compartmental model is shown as a flow diagram in Figure 4. Within this framework, women are classified according to their nursing period (number of pregnancies), from the proportion of pre-reproductive girls, *g*, to the proportion of women who, after a given number of pregnancies, *H*, have ceased to be reproductively active, *p*; the proportion of females in their *i-*th period of nursing are denoted by *n*_*i*_. Depending on transmission intensity, pregnant women contract PAM with probability *λ*, which can either be constant in time or variable to reflect seasonal fluctuation and/or epidemic outbreaks. It is assumed that after successful recovery women are protected against further episodes of PAM. The model can be described as the following set of differential equations:

(1)

with

(2)

In the model, *B *is the birth rate, 1/*μ *the average life expectancy, and *α *is the probability of dying as a result of PAM. It is assumed that girls become sexually (reproductively) active at an average age of 1/*m *and bear children at an average interval of 1/*c *years. To include temporal variations in transmission, *λ *was chosen to be generally time-dependent (as given in equation (2)), where *λ*_0 _is the background transmission between the main transmission seasons, *λ*_*s *_is the transmission intensity during a season or the epidemic peak, and *k *is a positive integer that inversely determines the duration of the transmission period (shorter for larger *k*). For simplicity, all other parameters are assumed to be constant in time and parity. Note, as malaria transmission is not explicitly model *per se*, the above system solely represents excess disease (and death) in the female population through PAM.

Within this framework, excess morbidity, *E*, is simply defined by the rate pregnant women move out of *g *and the susceptible classes *n*_*i *_multiplied by the probability of contracting PAM, *λ*; that is, , where *E*_*P *_and *E*_*M *_denote excess morbidity of the primi- and multigravidae classes, respectively. Excess mortality could be defined in exactly the same way and multiplying *E *by the probability of death as a result of PAM, *α*; however, as *α *is a circumstantial parameter it suffices to use *E *as a measure of the burden of PAM.

#### Stable transmission

**Figure 5:**
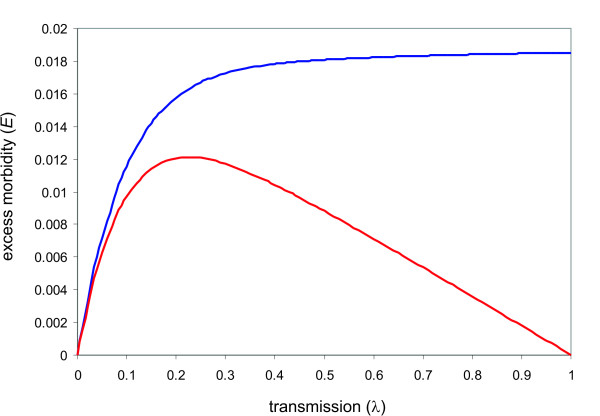
**The effects of transmission intensity on excess female morbidity and its contribution from multigravidae**. As the rate of transmission, *λ*, goes up, the excess female morbidity, *E *(blue line), initially increases but plateaus at medium levels of *λ*. At the same time, the contribution from multigravidae to excess morbidity (red line) declines as a greater proportion of women will contract PAM during their earlier pregnancies. Parameter values: *μ *= 1/40, *m *= 1/14, *c *= 1, *B *= 1/40, *α *= 0.1, *H *= 10.

Under the simplifying assumptions of constant birth and death rate and transmission intensity, the model converges towards a steady-state equilibrium (see Additional file 1), which allows us to directly analyse excess female morbidity, *E*, under changes in the level of transmission, *λ*. As shown in Figure 5, excess morbidity (blue line) expectedly increases as transmission intensifies but plateaus for medium to high levels of *λ*. At this point the contribution from multigravid women (red line) declines to zero and PAM is confined to the pool of women in their first pregnancies. Note, the maximum in excess morbidity in the multigravidae classes is not assumed at very low but at intermediate transmission levels. This is simply due to the fact that an initial increase in transmission inevitably affects all classes before it reaches a level where PAM is confined to women in their earlier and ultimately in their first pregnancies.

#### Unstable transmission

**Figure 6:**
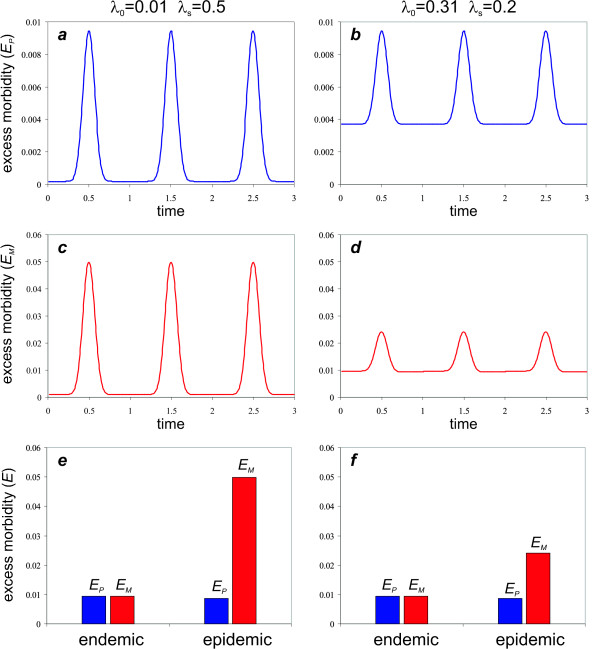
**Effect of seasonal transmission on excess morbidity in primigravidae and multigravidae at different background transmission rates**. The left panel illustrates a scenario of low background transmission between seasonal (or epidemic) outbreaks (*λ*_0 _= 0.01), whereas the right panel depicts a situation with moderate level of year-round transmission (*λ*_0 _= 0.2). The top row (*a*, *b*) shows the excess morbidity from primigravidae; the middle row (*c*, *d*) shows the excess morbidity of multigravid women, clearly indicating that higher background transmission significantly reduces excess morbidity from multigravid women during epidemic outbreaks. The bottom row (*e*, *f*) compares the maximum in excess morbidity from primi- and multigravidae (*E*_*M *_and *E*_*P*_, respectively) for the two background transmission rates with a stable transmission setting under equivalent maximum transmission rates, clearly demonstrating the effect of unstable transmission on multigravid women. Parameter values: *μ *= 1/40, *m *= 1/14, *c *= 1, *B *= 1/40, *α *= 0.1, *H *= 10, *k *= 10.

**Figure 7:**
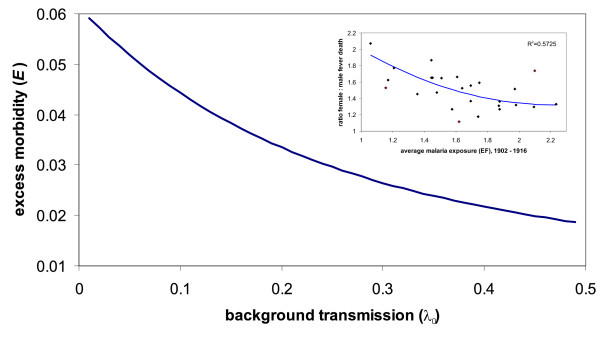
**The effect of prior exposure on PAM induced morbidity during epidemic outbreaks**. High year-round transmission results in most women experiencing PAM some time during their first pregnancies and offers a high degree of protection within the multigravidae class. Low levels of transmission on the other hand leave women of all parities vulnerable to the disease, causing a high degree of excess morbidity during large epidemics. Parameter values: *μ *= 1/40, *m *= 1/14, *c *= 1, *B *= 1/40, *α *= 0.1, *H *= 10, *k *= 10, *λ*_*S *_= 0.5-*λ*_0_.

To investigated how excess morbidity due to PAM is influenced by temporal variation in malaria transmission, the rate of transmission was decomposed into off- and on-peak levels, *λ*_0 _and *λ*_*s*_, respectively. Figures 6a-d demonstrate the result of varying the background transmission, *λ*_0_, on excess morbidity in primigravidae (6a and 6b) and multigravidae (6c and 6d) by keeping peak the transmission intensity *λ*_0 _+ *λ*_*s *_constant. Comparing the maximas in excess morbidity for the two different transmission settings (low and high level of background transmission) reveals that the peak in excess morbidity in primigravidae, *E*_*P*_, remains unaffected by a change in background transmission and is only determined by *λ*_0 _+ *λ*_*s*_. In contrast, multigravidae exhibit a significant reduction in peak excess morbidity when background transmission is higher. This can be explained by the inverse relationship between transmission intensity and the contribution of multigravidae to the excess in female morbidity. In other words, when transmission is low, a greater proportion of females go throughout their nursing stages unaffected by PAM and are thus fully susceptible during epidemic outbreaks. Importantly, excess morbidity during an outbreak significantly exceeds that of a comparable stable, endemic setting where *λ *= *λ*_0 _+ *λ*_*s*_. Figure 6e and 6f illustrate a direct comparison of excess morbidity in primi- and multigravidae, *E*_*P *_and *E*_*M*_, respectively, under the two different transmission scenarios. This clearly demonstrates that major epidemic outbreaks, which are preceded by a period of low transmission, will cause a considerably higher ratio in female to male morbidity/mortality than what can be expected in endemic regions with intense yet stable transmission. Therefore, not only does temporal variability of transmission change the signature of excess female morbidity compared to a year-round stable infection risk, it can also severely enhance the burden of PAM on the female population, especially when the level of exposure between epidemics is relatively low. Figure 7 illustrates the effect of varying the level of background transmission on the maximum in excess morbidity during seasonal/epidemic outbreaks and is in good qualitative agreement with the data from the Punjab region, shown as inset (same as Figure 3).

## Discussion

The adverse effects of malaria during pregnancy have long been recognized, but not until relatively recently have they gained much attention in terms of public health and intervention measures. It is clear from earlier work that malaria during pregnancy takes a different dynamic as the intensity of transmission changes through location and time [1,2,19]. There are also significant differences in its pathology and it is, therefore, useful to distinguish between placental malaria, or PAM, which specifically affects pregnant women and is caused by placental sequestration of *P. falciparum *parasitized red blood cells, and 'normal' malaria infections during pregnancy which can also, yet to a lesser degree, be caused by other Plasmodium species with adverse consequences for the mother and unborn child [20].

Placental malaria is the most common cause for low birth weight (LBW) in high transmission areas. It has been identified as the single greatest risk factor for neonatal mortality and contributor to infant mortality with an estimated 7-fold increase in the risk of still birth. Furthermore, it has recently been reported that children born to mothers with placental malaria also have an increased risk of malaria during their first few years of life [21]. In unstable and low transmission settings, on the other hand, malaria infections are more likely to cause clinical disease. Consequently, preterm delivery caused by febrile episodes is the biggest risk factor for low birth weight (LBW) in children in these regions. It appears, therefore, that in low endemic and unstable transmission settings mothers are the ones mostly affected by malarial infections during pregnancy, whereas in regions where transmission is intense, the burden is carried mainly by the foetus. However, women with symptomatic infections are more likely to seek out medical attention (if at all available), thus potentially reducing the risk of both severe disease and the establishment of placental malaria. This could lead to an underestimation of the risk of PAM in low and unstable transmission regions, and may account for the low measures reported in those settings [3,22,23].

Here, a method of using (historical) longitudinal data on excess female mortality has been used to qualitatively assess the impact of PAM on the female population in a malaria endemic area with considerable variations in transmission intensities. Although at this stage this can only be used as a proxy for the absolute level of mortality attributable to PAM, it clearly indicates a significant contribution of pregnancy-associated malaria to female mortality, especially during epidemic outbreaks. This approach is based on the observation that the ratio of female to male mortality is not constant over time but shows a strong correlation with transmission intensity. That is, if extra female deaths were simply due to complications during labour and/or infections during pregnancy, this number would expectedly be relatively constant and independent of the intensity of malaria transmission. In addition, excess female mortality is negatively and (possibly) non-linearly correlated with prior exposure to malaria as would be expected if it were attributable to PAM. Finally, the significant increase in excess mortality during an epidemic outbreak, compared to non-epidemic years, clearly demonstrates the existence and damaging effect of pregnancy specific malaria in a region where transmission is moderate and unstable.

A simple mathematical model demonstrates how overall female morbidity may be expected to increase with transmission intensity while shifting the contribution from women of all gravidity towards those in their first pregnancy. In low transmission settings there is a chance that women reach a non-reproductive stage without ever contracting PAM, whereas in hyper-endemic regions most women will contract the disease during their first pregnancy. Interestingly though, it was found that the contribution from multigravidae to excess female morbidity is not linear but peaks at low to medium levels of transmission and, as expected, diminishes towards to upper end of the endemicity spectrum. It is also clear from this analysis that the total average number of pregnancies impacts the magnitude of morbidity among females, at least in regions of low and unstable malaria (see Additional file 1). Thus, a reduction in the average number of pregnancies (for example through education on birth control) could have an impact on mortality in areas where transmission intensity is moderate but would have little effect in hyperendemic situations. This may, however, be altered by the spread of HIV as recent reports suggest that HIV infection can impair humoral immunity to placental type VSA [24,25]. If so, the age structure (and overall incidence) of PAM could shift in a direction, even in hyperendemic areas, that would warrant intervention through birth control.

The greatest effect of PAM on the female population observed within this framework was by incorporating seasonal variations in transmission intensity. Given sufficiently low background transmission rates, many women will go through successive pregnancies without getting infected, leaving a significant proportion of the reproductively active female population still susceptible to PAM. As a result, and in accordance with the data presented here, the observable excess in female morbidity during epidemic outbreaks is significantly higher than within a stable transmission setting and equivalent transmission intensity, and even more so as the level of transmission in the period preceding an epidemic is especially low.

These data also support the current conception of PAM as being particularly severe among females with low exposure to non-PAM malaria as compared to those living in hyperendemic areas. There are a number of reasons why this may be the case. In hyperendemic areas, women of childbearing age will typically have protective antibodies to the other PfEMP1 proteins encoded by the *var *genes of parasites expressing placental binding *var2csa*, and this may protect them from disease while allowing infection of the placenta (which still has severe consequences for the foetus); in areas of low transmission, women of childbearing age may not have immune responses against the other PfEMP1 proteins encoded by the infecting parasite and thus may experience disease as well as infection. Individuals living in areas of high transmission may also have antibodies to VAR2CSA itself without having sustained a placental infection [26], although it remains to be shown if the antibody levels reported are protective against disease. Finally, there are of course a very large range of other immune responses that could prevent an outcome of severe complicated malaria upon infection with parasites expressing placental binding *var2csa*. However, as men and women alike will lack these forms of protective immunity in areas of low transmission, the excess mortality attributed to PAM strongly implies that placental infection itself can greatly increase the chances of death among individuals with incomplete immunity. Indeed, if immunity to severe disease is acquired upon one or two infections [7], the analyses presented in this paper indicates that this form of immunity clearly does not extend to PAM. The unusually conserved nature of VAR2CSA makes it an attractive vaccine candidate, but until this vaccine becomes available, ongoing and future prevention measures, such as intermittent preventive treatment (IPTp) against malaria, in particular during pregnancy (see e.g. [27,28]), have to be urgently employed not only in regions of extensive levels of transmission but equally in regions where malaria is more sporadic.

## Competing interests

The authors declare that they have no competing interests.

## Authors' contributions

MR, SG, PB and APD devised and analyzed the model; MJB provided and analyzed the data and contributed to the writing of the manuscript. MR, APD and SG wrote the manuscript.

## Supplementary Material

Additional file 1 **Model analysis**. The supplementary material presents further analysis of the model's behaviour under stable transmission conditions and derives an analytical estimate of the maximum in excess morbidity in the multigravid class. Click here for file
