# Supplementary material for: Assessing the burden of pregnancy-associated malaria under changing transmission settings
Source: Malar J. 2009 Oct 28;8:245. doi: 10.1186/1475-2875-8-245 (PMC2774336; doi:10.1186/1475-2875-8-245)
Supplement: Additional file 1 — Model analysis. The supplementary material presents further analysis of the model's behaviour under stable transmission conditions and derives an analytical estimate of the maximum in excess morbidity in the multigravid class. [file 1475-2875-8-245-S1.pdf]

SUPPLEMENTARY MATERIAL

Assessing the burden of pregnancy associated malaria under changing transmission settings

Mario Recker[1], Menno J Bouma [2], Paul Bamford[1], Sunetra Gupta[1§], Andy P Dobson[3]

Steady state under stable disease transmission (λ_s=0)

Assuming a constant birth rate *B*, stable transmission (λ_s=0) and ignoring other demographic factors, system (1) converges towards a steady-state equilibrium given by:

$$G := N_0 = \frac{B}{m+\mu}, \quad N_i = mc^{i-1}\left(\frac{1-\lambda}{c+\mu}\right)^i N_0,$$
$$R_i = \frac{cm\lambda(1-\alpha)}{c+\mu}\sum_{j=0..i}\left(\frac{c}{c+\mu}\right)^{i-j} N_j, \; P = \frac{c}{\mu}\left(R_H + (1-\alpha\lambda)N_H\right), \quad (2)$$

where capital letters denote equilibrium values. Within this framework we define excess morbidity, *E*, simply by the rate pregnant women move out of the susceptible classes *g* and n_i multiplied by the probability of contracting PAM, λ; that is

$$E = E_P + E_M \quad \text{with} \quad E_P = m\lambda G, \quad E_M = \sum_{i=1..H} E_i = \sum_{i=1..H} c\lambda N_i \quad (3)$$

where E_P and E_M denote excess morbidity of the primi- and multigravidae classes, respectively.

Maximum in excess morbidity in the multigravidae classes

From Figure 5 (main text) it is clear that the excess morbidity within the multigravidae class assumes a local maximum. From (3) and (4) we find $E_i^{\max}$ exactly when:

$$\frac{\partial}{\partial\lambda}E_i = \frac{\partial}{\partial\lambda}\left(c\lambda N_i\right) = 0 \quad \Leftrightarrow \quad \lambda_i^{\max} = \frac{1}{1+i}. \quad (4)$$

($\lambda = 0$ and $\lambda = 1$ are the local minima.) This means that the highest impact of PAM on senior gravid women occurs at lower levels of transmission than on women in earlier pregnancies. And because $E_i^{\max}$ is a decreasing function in *i*, the total excess in morbidity from multigravidae, $E_M^{\max}\left(= \sum_{i=1..H} E_i^{\max}\right)$, is observed at intermediate levels of transmission intensity. Unfortunately, the exact value of $\lambda^{\max}$ requires solving a polynomial of degree *H* but we can find a good approximation in:

$$\lambda^{\max} \approx \sum_{i=1..H} a^i \lambda_i^{\max} / \sum_{i=1..H} a^i \ , \qquad (5)$$

with $a = c/c + \mu$. As such, $\lambda^{\max}$ depends on the average number of pregnancies per female, *H*, and, to a smaller degree, on the duration of the period between consecutive pregnancies, *c*. Therefore, as the lifetime number of pregnancies increases, the maximum impact of PAM induced excess morbidity occurs at lower transmission intensities.
